# Partial Hydatidiform Mole in an Ectopic Tubal Pregnancy

**DOI:** 10.7759/cureus.15455

**Published:** 2021-06-05

**Authors:** Abdulkarim Hasan, Ahmed Elhawary, Mohamed F Abdelaleem, Tarek Hegazy, Khalid M Nafie

**Affiliations:** 1 Pathology, Faculty of Medicine, Al-Azhar University, Cairo, EGY; 2 Primary Health Care Centers, Ministry of Health, Taba, EGY; 3 Primary Health Care Centers, Ministry of Health, Cairo, EGY; 4 Obstetrics and Gynaecology, Ministry of Health, Tanta, EGY; 5 Laboratory and Blood Bank, Prince Mishari Bin Saud Hospital, Ministry of Health, Baljurashi, SAU

**Keywords:** surgical acute abdomen, ruptured ectopic pregnancy, histopathology examination, hydatidi form moles, fallopian tubes

## Abstract

Fallopian tubal molar pregnancy is extremely rare, and the main diagnostic tool is the post-operative histopathological diagnosis, as the pre-operative diagnosis is difficult. We report a case of ectopic partial molar pregnancy in the right fallopian tube of a 35-year-old lady that was sent for routine histopathological examination with the clinical diagnosis of ectopic pregnancy and the histopathology report revealed an unusual result.

## Introduction

Gestational trophoblastic diseases (GTDs) consist of placental site trophoblastic tumor, choriocarcinoma, epithelioid trophoblastic tumor, and hydatidiform mole. The majority of GTDs occur in the uterus, but ectopic molar pregnancy is extremely rare (ectopic GTD incidence is 1.5 per one million births in the UK) [1]. There are two types of molar pregnancy: partial and complete moles. The partial molar pregnancy may be associated with a fetus [2]. The diagnosis of molar pregnancy at ectopic sites is very difficult as there is no distinguishing clinical feature but depends mainly on the pathology and DNA ploidy analysis [3]. Although molar pregnancy and ectopic pregnancy are not rare events, the combination of the two, an ectopic molar pregnancy, is an extremely rare event [4]. Here, we present a case of tubal partial hydatidiform mole that was treated with salpingectomy and accidentally discovered during the histopathological examination, and the case possessed a favorable prognosis.

## Case presentation

A 35-year-old female came to the primary care center complaining of acute lower abdominal pain with a history of amenorrhea for two months and spotting per vaginum. Upon presentation, the abdominal pain was of sudden onset, not radiating, continuous, and not relieved by oral analgesics. There was no history of vomiting or fever. Her blood pressure (arterial blood pressure, ABP) was 105/75 mmHg, with a pulse of 70 beats/minute. Clinical examination revealed a soft abdomen and mild tenderness in the right iliac fossa.

A pregnancy test was requested and revealed a positive result with elevated human chorionic gonadotropin β (β-hCG) titer (30000 units/mL). The patient was referred to the obstetric department at the referral hospital, in which she reported an obstetric score of gravida 3 para 2a with normal past obstetric history. Per vaginal examination revealed right cervical tenderness. Pelvic ultrasonography showed a 4.5 cm x 3 cm swelling adjacent to the right ovary with a gestational sac containing a viable gestational sac with active heartbeats and body motions, a gestational age of 10 weeks, and a uterus within the normal size. Hemoglobin level was 10.5 g/dL, other routine hematology and biochemistry examinations were within normal ranges.

The patient was primarily diagnosed clinically with ruptured ectopic pregnancy. Explorative laparoscopy was performed and a ruptured mass was detected in the ampulla of the right fallopian tube. A little free fluid collection in the Douglas pouch was also seen. Right salpingectomy was performed and sent to the histopathology laboratory in a formalin fixative fluid.

**Figure 1:**
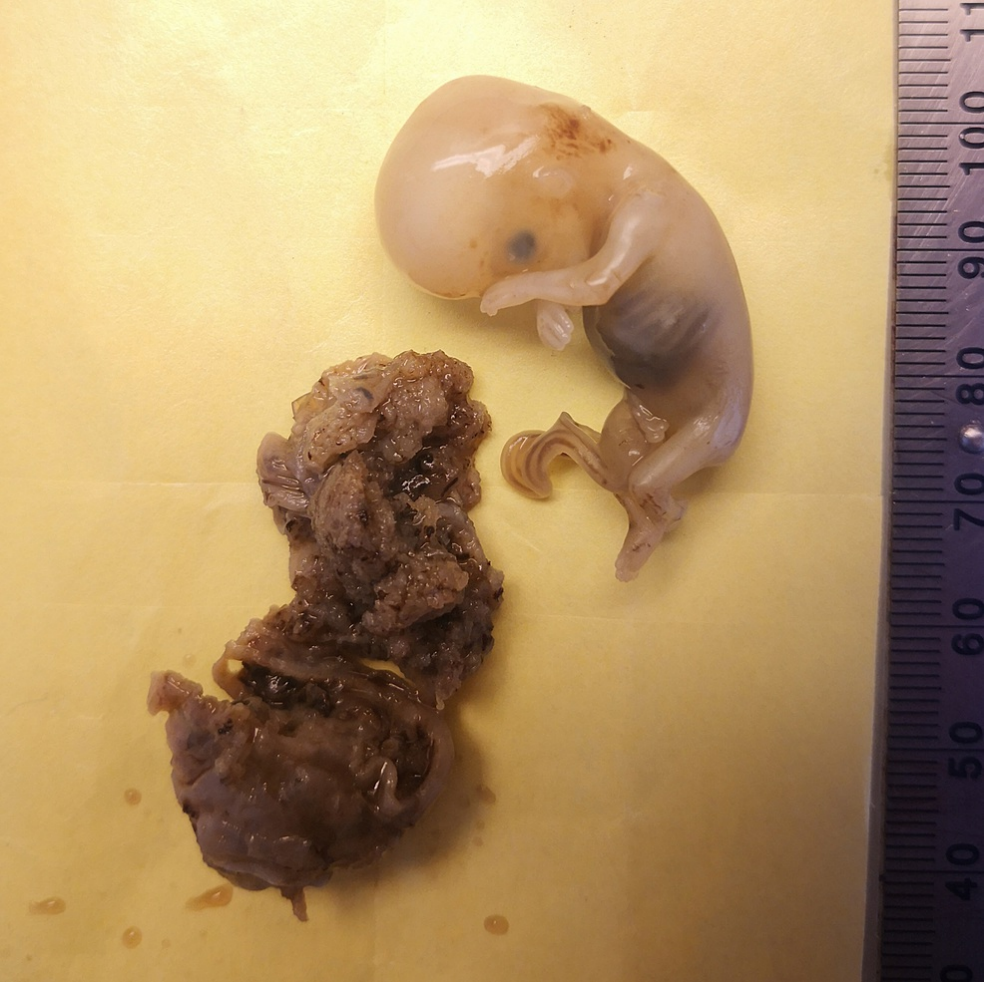
Gross picture. A gross picture showing ruptured fallopian tube, blood clots, small vesicles, and fetal tissue

**Figure 2:**
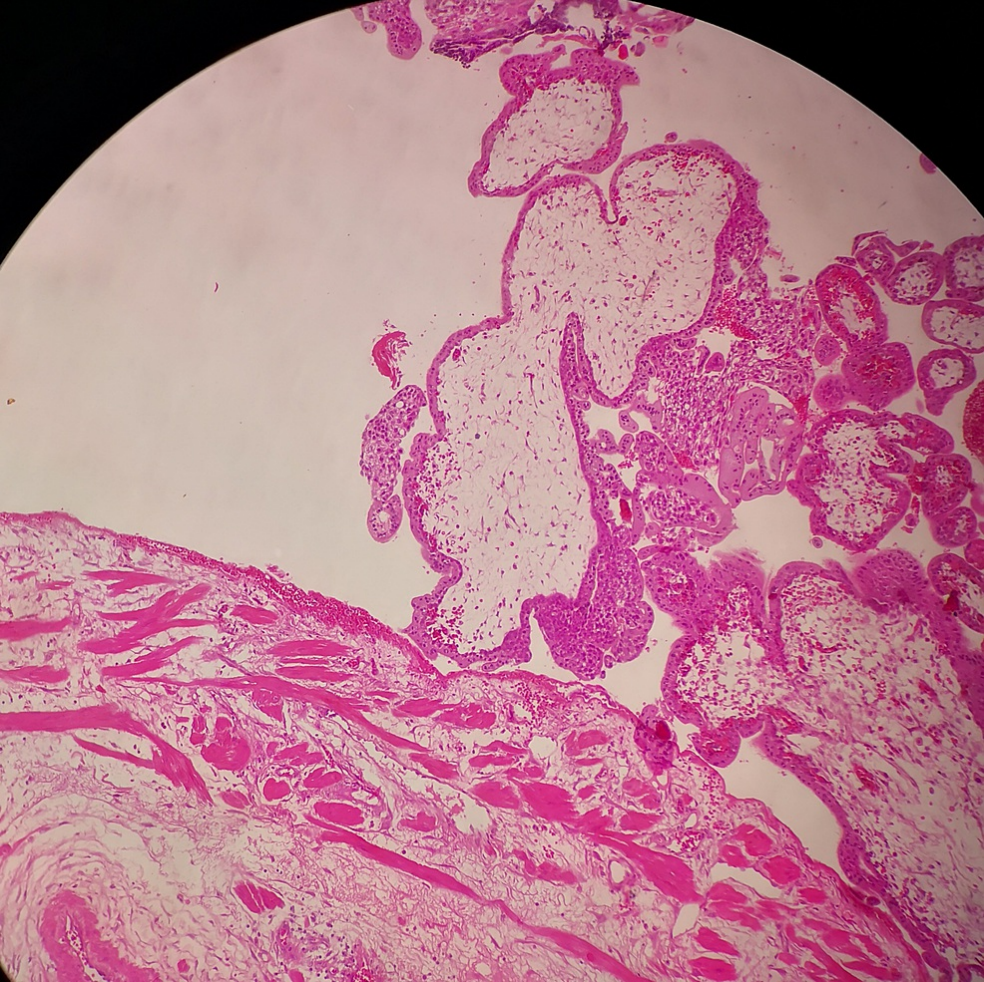
Microscopic picture. A microscopic picture showing edematous villi along with villi with polar trophoblastic proliferation attached to the wall (200x, H&E).

Grossly, the content of the fallopian tube consisted of a lump of blood clots in which edematous-looking villi and fetal tissue measuring 4.5 cm were seen. Upon microscopic examination by the senior pathologists, polar proliferated syncytiotrophoblastic and cytotrophoblastic cells were noted with irregular shape and edematous stroma with fetal tissue. The hydropic appearance was mild to moderate. Histological features are consistent with a non-invasive molar pregnancy, the complete mole was excluded due to the presence of fetal parts, the patient was advised for karyotype analysis, and the final diagnosis was confirmed as a partial hydatidiform molar pregnancy. Follow-up with β-hCG serum level was done and a gradual decline of the hormone level was recorded without noted complications (Figures 1-2).

## Discussion

Tubal molar pregnancy is an extremely rare event. The incidence of the disease is estimated to be 1-5/200,000 which considerably varied in different countries [5].

The diagnosis of this disease and several other rare gynecological and obstetrician diseases depends mainly on histopathology studies, however, it is difficult histologically to be distinguished as molar from non-molar hydropic changes [6-9].

Hydatidiform molar pregnancy is characterized by hydropic degeneration of chorionic villi associated with the proliferation of trophoblasts due to fertilization of the abnormal ovum. In complete mole, the genome is entirely made of paternal origin, due to fertilization of an empty ovum by a haploid sperm followed by chromosomal duplication or by fertilization by diploid sperm. Partial hydatidiform mole is a pathological process due to fertilization of the ovum by two sperms making a paternal to maternal chromosome ratio of 2:1 [10]. There was no reported definite risk factor for ectopic molar pregnancy owing to the scarcity of the disease, however, there are many suggested risk factors for abnormal pregnancies including pelvic inflammatory disease, intrauterine contraceptive devices, tubal surgery, and a previous ectopic pregnancy [11].

Clinically tubal molar pregnancies are usually indistinguishable from non-molar ectopic pregnancies. β-hCG levels are also not useful in differentiation [5, 12]. All surgically removed ectopic pregnancies depend on histopathological examination to specify or exclude molar change. Molar should be distinguished by the non-molar pregnancy as the former can cause a persistent trophoblastic disease or even metastasis to distant organs. Hence close follow-up is essential after the removal of products [4]. In difficult cases, the DNA flow cytometry for ploidy examination and P57 immunocytochemical staining is useful for differentiating partial from complete mole [13]. In our case, the microscopic features are inconsistent with the complete hydatidiform mole due to the presence of the fetal parts.

During the histopathological examination, pathologists should keep in their minds that, compared with evacuated intra-uterine products of conception, the degree of extravillous trophoblastic proliferation might appear more florid in the ectopic gestation [7, 13]. Laparoscopy is the main way for the treatment of ectopic pregnancy cases. Pasic et al. have suggested that surgical treatment for the majority of patients should be salpingotomy in case of lack of rupture [14]. Our case was a ruptured tube at the time of diagnosis that is why the salpingectomy was the treatment of choice.

## Conclusions

However, it is a very rare occurrence; ectopic molar pregnancy should be considered as a possibility in women presenting clinically with a suspected ectopic pregnancy. The histopathological examination of the salpingectomy specimen and conceptus in tubal pregnancies is essential for accurate diagnosis and appropriate follow-up management and histopathologist doctors should keep in minds the rare occurrence of molar proliferation in ectopic tubal pregnancies.

.
